# Blood feeding tsetse flies as hosts and vectors of mammals-pre-adapted African *Trypanosoma:* current and expected research directions

**DOI:** 10.1186/s12866-018-1281-x

**Published:** 2018-11-23

**Authors:** Anne Geiger, Imna Malele, Adly M Abd-Alla, Flobert Njiokou

**Affiliations:** 1 INTERTRYP, Institut de Recherche pour le Développement, University of Montpellier, Montpellier, France; 2 Vector and Vector Borne Diseases Institute, Majani Mapana, Off Korogwe Road, Box, 1026 Tanga, Tanzania; 3 Insect Pest Control Laboratory, Joint FAO/IAEA Division of Nuclear Techniques in Food and Agriculture, Vienna, Austria; 4 Faculty of Science, University of Yaoundé I, P.O. Box 812, Yaoundé, Cameroon

**Keywords:** Vector control, Tsetse flies, Bacteriome, Trypanosomes

## Abstract

Research on the zoo-anthropophilic blood feeding tsetse flies’ biology conducted, by different teams, in laboratory settings and at the level of the ecosystems- where also co-perpetuate African *Trypanosoma-* has allowed to unveil and characterize key features of tsetse flies’ bacterial symbionts on which rely both (a) the perpetuation of the tsetse fly populations and (b) the completion of the developmental program of the African *Trypanosoma*. Transcriptomic analyses have already provided much information on tsetse fly genes as well as on genes of the fly symbiotic partners *Sodalis glossinidius* and *Wigglesworthia,* which account for the successful onset or not of the African *Trypanosoma* developmental program. In parallel, identification of the non- symbiotic bacterial communities hosted in the tsetse fly gut has recently been initiated: are briefly introduced those bacteria genera and species common to tsetse flies collected from distinct ecosystems, that could be further studied as potential biologicals preventing the onset of the African *Trypanosoma* developmental program. Finally, future work will need to concentrate on how to render tsetse flies refractory, and the best means to disseminate them in the field in order to establish an overall refractory fly population.

## Background

**Fig. 1:**
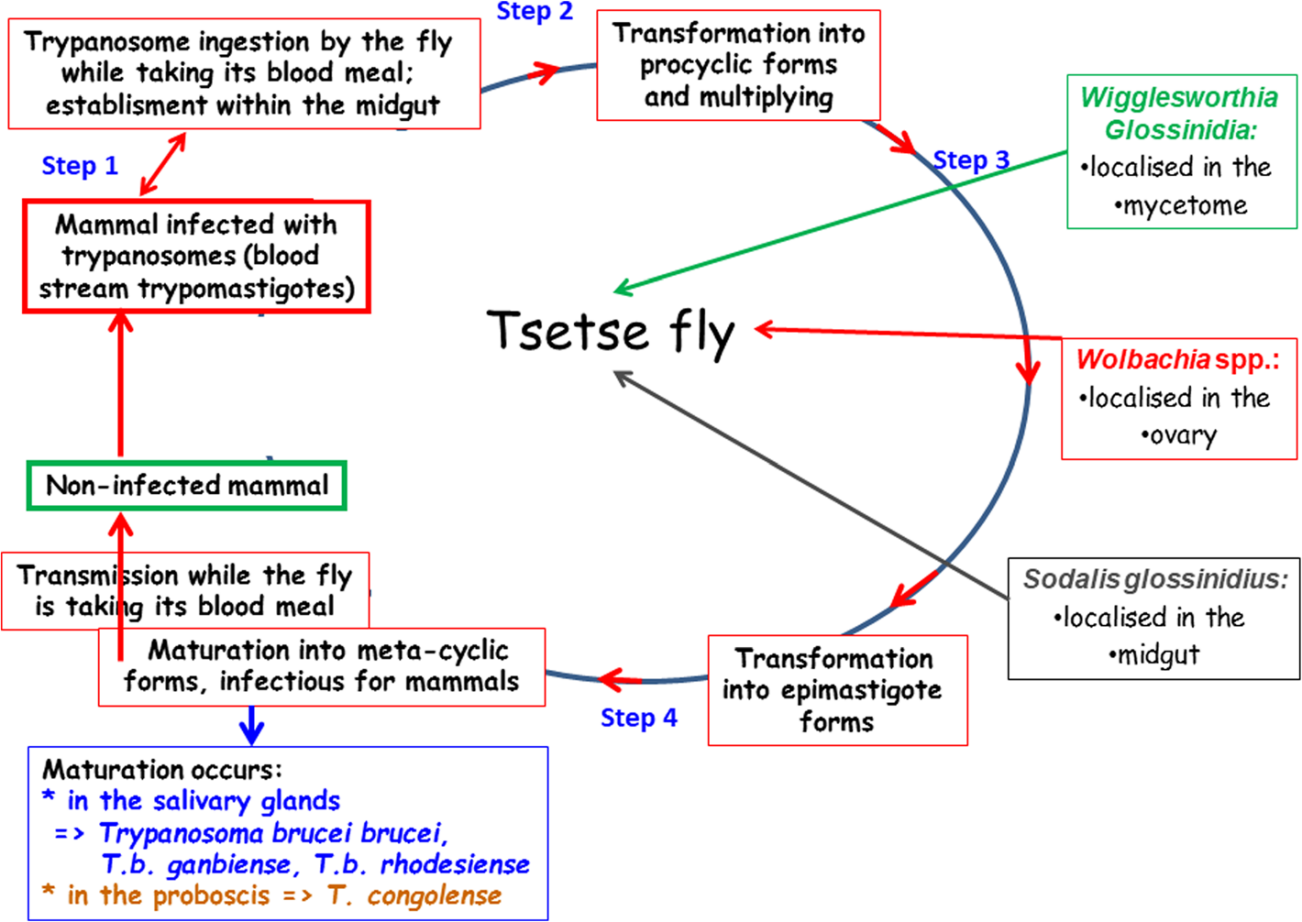
Tsetse fly showing localization of its three symbionts and the trypanosome life cycle into the fly

Human African trypanosomiasis (HAT) and animal African trypanosomiasis (AAT or nagana) are caused by flagellate protozoa belonging to the genus *Trypanosoma*. The parasites are transmitted to the vertebrate host (most typically a mammal) by a hematophagous insect, the tsetse fly, in which the parasites complete part of their life cycle (Fig. 1). In terms of mortality, HAT is ranked ninth out of 25 human infectious and parasitic diseases in Africa. Despite its severity, HAT is one of the most neglected tropical diseases [1].

Two subspecies, *Trypanosoma brucei gambiense* and *Trypanosoma brucei rhodesiense*, are respectively transmitted by the tsetse flies *Glossina palpalis* and *Glossina morsitans* and both are pathogenic to humans. *T. b. rhodesiense* causes the acute form of sleeping sickness in East Africa, which can lead to patient death within a few weeks if left untreated, whereas *T. b. gambiense* is responsible in Central and West Africa for the chronic form of the disease that develops gradually over several months to several years. The serious nature of this disease has led to it being targeted for elimination by both the WHO and the Pan-African Tsetse and Trypanosomiasis Eradication Campaign (PATTEC), and subsequently by the London Declaration on Neglected Tropical Diseases [2–5].

Recently, the number of newly diagnosed cases has begun to decrease, a situation that occurred in the 1960s that preceded the last heavy outbreak in the 1990s. Thus, despite this decrease in the detection of new cases, the severity of the situation requires continuing research to improve our current approaches (both in terms of treatment and diagnosis), as well as the development of novel control strategies that can be alternative or complementary to currently used anti-vector methods (such as mobile and stationary traps, release of sterile males). Indeed, reducing fly populations or their vector competence (i.e. rendering the fly refractory to trypanosome infection) or even the delivery of in situ trypanocidal compounds could reduce or suspend parasite dissemination, and consequently, the spread of the disease.

**Fig. 2:**
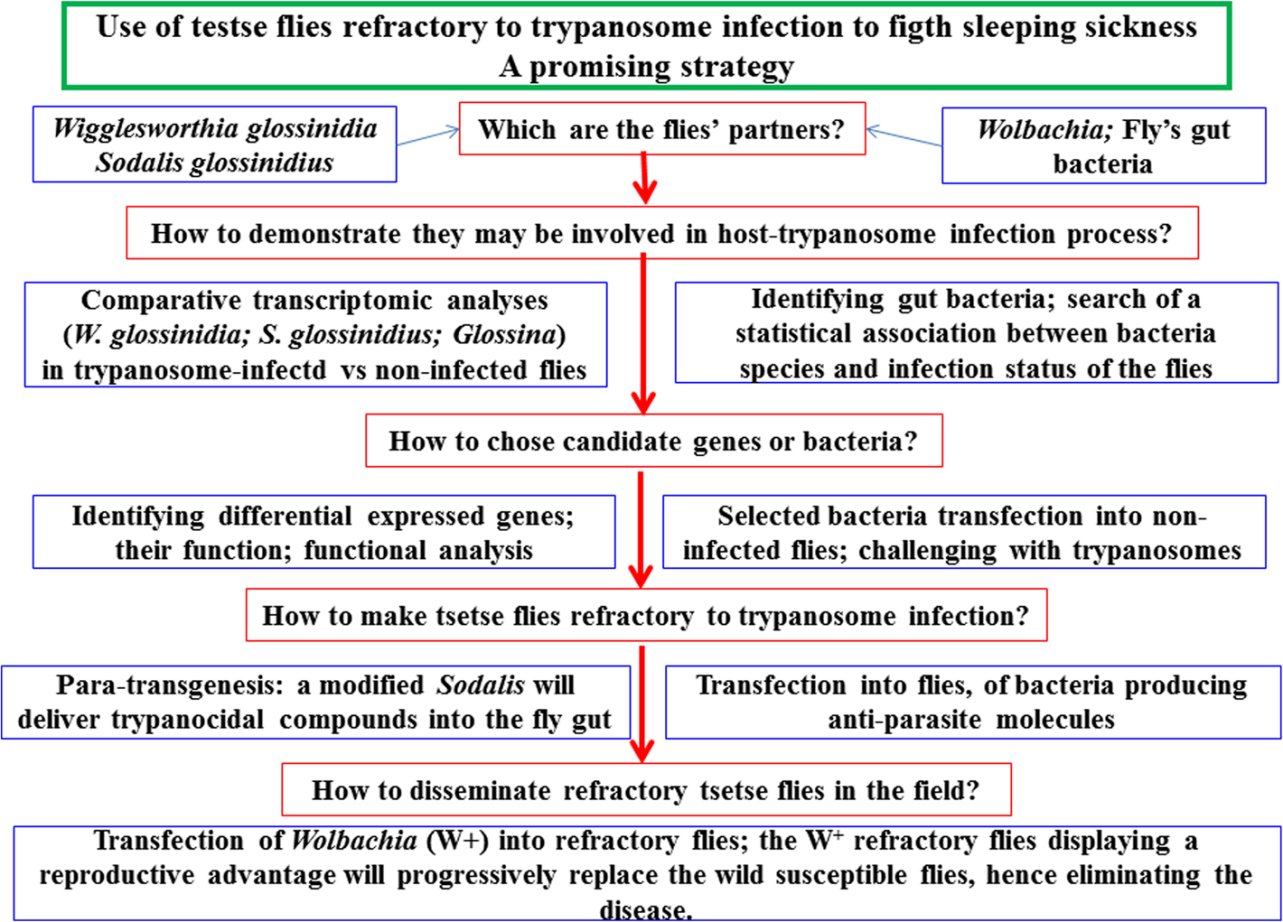
Summary of the main steps involved in the “refractory fly” strategy to control sleeping sickness. Several steps (such as transcriptomic analyses, identifying of differentially expressed genes, identifying of gut bacteria from field flies) have been performed or are in progress. Similarly, technical aspects (choice of a promoter for an efficient transgene expression in *Sodalis*, *Sodalis* transfection into tsetse flies) are being solved

The successful completion of this objective requires identifying potential targets, with the caveat that some targets that are experimentally promising under laboratory conditions may not be applicable in the field operations. Included among these possible targets are the microorganisms that comprise the tsetse fly microbiome, since they likely interact with each other as well as with their host and the trypanosome and thus may contribute, positively or negatively, to tsetse infection. Furthermore, this approach will require the characterization of the mechanisms (particularly molecular) involved in these interactions, which will also necessitate determining how to stimulate or repress these mechanisms (depending on the objective). Finally, this will require testing and selecting protocols that can result in an effective, efficient, and environmentally safe field application. Here, we review these different elements (summarized in Fig. 2), some of which are already the subject of active research projects.

## Blood-feeding tsetse fly symbiotic and non-symbiotic bacteria communities and their potential use as biologicals to prevent the onset of African *Trypanosoma* developmental program

### A brief overview about the early and complex tissue milieu where is initiated or not the African *Trypanosoma* developmental program

The unfolding or not of the pool blood feeding African *Trypanosoma* developmental program in the pool blood- feeding tsetse flies start when they sample their blood meal from mammals hosting tsetse fly preadapted developmental stage population: the latter populations – either *Trypanosoma brucei gambiense* or *Trypanosoma brucei rhodensiense* – or *Trypanosoma congolense* being delivered within the blood meal in the posterior part of the tsetse fly midgut. [6–11]. The parasites undergo proliferation and differentiation during this migration, ending in the metacyclic form, which is the only infectious form to the mammalian hosts.

The trypanosome infection rate in tsetse fly (whether natural or experimental) is low, indicating that most individuals within a fly population might be refractory to parasite infection [12–15]. In fact, the success of infection (that starts the trypanosome life cycle within its host) depends on the parasite’s capacity to circumvent the tsetse immune responses. This success also depends on the host’s ability to respond to the parasite’s attempts to breach its first line of defense (for example by the production of trypanocidal compounds). This “game” of offensive and counter-offensive maneuvers is diverse and only ends when one of the participants takes the upper hand. Specifically, if the parasite ‘wins’, then it can complete its cycle and the tsetse fly performs its role as vector to transmit the trypanosome to the mammalian host. In contrast, if the fly ‘wins’, then this “refractory” fly will eliminate the parasite.

Though in the above § we got recourse to the war game metaphor, we like considering the more biologically relevant context of the unfolding or not of the African *Trypanosoma* developmental program in the tsetse fly holobiont. Such a context allows framing the *Trypanosoma* blood feeding -tsetse fly interactions within the context of those inter-organ communications that shape their physiology its immune and metabolic components included. The metazoan gut microbiome is increasingly recognized to play an important role in shaping and maintaining the health of the studied metazoans. Among the model fly with which to further feed this theme, *Drosophila* is offering potential to increase our understanding of how the microbiome influences tsetse flies’ traits such as the immune system sensors and the metabolism networks [16–19].

A number of fly defense mechanisms have been reported that are involved in fly resistance to trypanosome infection, including innate immune responses, the production of reactive oxygen and antimicrobial compounds, the involvement of lectins, and Immune Deficiency (Imd) pathway-regulated gene expression [20–26]. Conversely, “silencing” Glutamic Acid – Proline (EP) protein will favor the trypanosome infection [20]. In fact, it was suggested the (EP) protein to act as a lectin [27] agglutinating trypanosomes. In addition, it was demonstrated that tsetse EP protein was strongly up regulated following stimulation with *Escherichia coli* [28] providing evidence that it is part of the immune response of the flies. However, the precise mechanism of its action on vector refractoriness needs still to be elucidated. Another fly defense is the proventriculus, an active insect immune tissue [29] that challenges the trypanosome transmission to the salivary glands (few trypanosomes can pass through it), where they undergo differentiation to the metacyclic form. Along Hao et al. [22] microbial challenging of tsetse flies stimulates the proventriculus production of not only antimicrobial peptides (attacin and defensin) but also of reactive nitrogen and oxygen intermediates that in turn may be responsible for the activation of other immune genes involved in refractoriness. Examples cited above are the fly’s “weapons”, but little is known about how trypanosomes circumvent the immune response during the various stages of their development in tsetse tissues and organs; thus trypanosome’s “weapons” still need to be clarified.

### The blood-feeding tsetse fly symbiotic and non-symbiotic bacteria communities hosted within their gut: Their impact on the onset or not of the developmental program of African *Trypanosoma*

For a long time, only three bacteria species were identified as inhabitants of the tsetse fly gut: the obligate symbiont *Wigglesworthia glossinidia* (Enterobacteriaceae), and the facultative symbionts *Sodalis glossinidius* (Enterobacteriaceae) and *Wolbachia* (Rickettsiaceae). However, recently, more systematic investigations using either culture-dependent or molecular (culture-independent) approaches have been performed to demonstrate that the fly microbiome is far more rich and diverse, as it will be shown below. Although the interactions between the microbial symbionts and their insect host (including their possible interference with the host’s susceptibility to trypanosome infection) have been actively studied in the past, these types of investigations are almost absent today regarding recently identified bacteria from the intestinal flora.

### Wigglesworthia glossinidia

All *Glossina* flies harbor the obligatory symbiont *Wigglesworthia* [30, 31]. This symbiont is localized intracellularly in specialized host cells (bacteriocytes) in the anterior midgut forming the bacteriome [32]. An additional extracellular population of *Wigglesworthia* is also found in milk glands of the female fly, allowing maternally transmission of the bacterium to the fly’s offspring [33]. In contrast to the tsetse fly, the enzymes of several vitamin biosynthetic pathways are encoded by *W. glossinidia,* thus, the main role of this symbiont within the fly is to produce and provide its host with vitamins that are normally absent from the tsetse blood diet [34–37]. *Wigglesworthia* (Wgm) also has an immunomodulatory effect in tsetse. Specifically, *Glossina morsitans morsitans* flies devoid of the symbiont were shown to be immunodepressed as compared to wild flies [36, 38, 39]. Since the symbiont is involved in fly immunity, it also participates in its susceptibility to trypanosome infection. The host’s peptidoglycan recognition protein LB (PGRP-LB), a catalytic member of the PRGP family [40], which cleaves bacterial peptidoglycan into non-immunogenic fragments thus preventing stimulation of the fly’s Imd pathway which otherwise would have a deleterious effect on maintaining the fly’s symbiosis with Wgm [38, 41]. It must be noted that PGRP-LB expression is *Wigglesworthia*-dependant. Since PGRP-LB also has trypanocidal activity, increased concentrations of this protein could result in the increased refractoriness of tsetse flies to trypanosome infection, in non-teneral tsetse flies.

### Sodalis glossinidius

*S. glossinidius* is a commensal symbiont found in all insectary-raised tsetse flies [42–44]. This bacterium is vertically transmitted through the milk gland secretions of the female tsetse to its offspring. *Sodalis* displays a wide tissue tropism, and is found intracellularly and extracellularly in the tsetse intestine, hemolymph, and salivary glands [33].

This symbiont is of great interest to the tsetse fly infection mechanism, since epidemiological surveys in several HAT foci indicate that *Sodalis* favors fly infection by trypanosomes [45]. In addition to the observation that *Sodalis* populations are not genetically homogeneous, it has been reported that: the genetic structure of these populations depends on the tsetse species host [43]; the population structure could differ between HAT foci [46]; and finally, there is a relationship between a given symbiont genotype and the fly infection with a given trypanosome species [44]. These results demonstrate the complex genetically-based association between the symbiont and the trypanosome infection in tsetse fly, which governs (or at least modulates) the vector competence of the *Glossina* fly and thus the spread of sleeping sickness. This indicates that *Sodalis* has potential as a target for vector control.

### Wolbachia

The symbiotic bacterium *Wolbachia* has been reported to infect approximately 40% of all arthropods [44–49]. It is localized intracellularly in the reproductive organs, and primarily transmitted vertically by the female to her offspring [31]. *Wolbachia* generates reproductive abnormalities in the infected host, such as cytoplasmic incompatibility (CI), feminization, and parthenogenesis; it can also intervene in host fertility, immunity, longevity, and development [50–53]. Tsetse populations host genetically distinct but closely related *Wolbachia* strains [54, 55]. It is particularly interesting that *Wolbachia* generates strong CI in tsetse flies, as in other arthropods [56]. Later in this review we will present how these particular interactions that the symbiont establishes with its host can be exploited as part of a vector control strategy against sleeping sickness, or possibly against other vector-borne diseases.

### Microbiota bacteria, other than Sodalis, Wigglesworthia and Wolbachia

#### Identification and diversity

Investigations into the possible presence of bacteria other than the three symbionts described above in the tsetse fly gut have only recently begun. In fact, most work has focused on the fly microbiome composition, following previous results on the role of the symbionts in tsetse infection with trypanosomes. This raises the issue: if symbionts have a role in the infection process, are any other gut bacteria implicated as well? Geiger et al. used bacterial isolation and culture-dependent approaches to isolate and identify a novel bacteria species (*Serratia glossinae*) in insectary-raised *Glossina palpalis gambiensis* flies [57], and several bacteria species from the gut of different *Glossina* species that were sampled in Angola and Cameroon [58, 59]. Most of the bacterial species belonged to the *Enterobacter*, *Enterococcus*, and *Acinetobacter* genera, whereas others (from flies sampled in Cameroon) belonged to *Providencia*, *Sphingobacterium*, *Chryseobacterium*, *Lactococcus*, *Staphylococcus*, and *Pseudomonas*. Similar investigations were performed on *Glossina fuscipes* flies sampled in Kenya [60] and Uganda [61], using either culture-dependent or molecular approaches (i.e. deep sequencing of the V4 hypervariable region of the *16S rRNA* gene) to identify a large number of bacterial genera. Finally, using similar approaches, a related study is currently in progress on *Glossina pallidipes* flies sampled from Tanzania (Malele I, Nyingilili H, Lyaruu E, Tauzin M, Ollivier B, Fardeau M-L, Geiger A: Bacterial diversity in the gut of G. pallidipes population from a non-sleeping sickness focus in Tanzania and its implication for species’ vectorial capacity, in preparation), in which bacteria from the genera *Bacillus*, *Acinetobacter*, *Mesorhizobium*, *Paracoccus*, *Microbacterium*, *Micrococcus*, *Arthrobacter*, *Corynobacterium*, *Curtobacterium*, *Vagococcus*, and *Dietzia* have been identified.

#### Could the non-symbiotic bacteria be genetically engineered to prevent the tsetse flies to act as hosts and vectors of African Trypanosoma?

As the first stage of trypanosome infection takes place in the gut of tsetse flies, the discovery of these gut bacteria and their potential use as targets for vector control clearly represents an exciting future. In fact, a number of the isolated bacteria are reported to affect insect survival and/or vector competence (such as in: *Anopheles albimanus* / *Serratia marcescens* / *Plasmodium vivax*; *Anopheles funestus* / Gram-positive bacteria / *Plasmodium falciparum*; *Rodnius prolixus* / *S. marcesens* / *Trypanosoma cruzi*) [62]. The production of anti-parasitic molecules by bacteria species similar to those present in the tsetse fly gut has been frequently reported, including cytotoxic metalloproteases produced by *S. marcescens* and *Pseudomonas aeruginosa* [63], or hemolysins secreted by *Enterobacter* spp., *E. coli*, *S. marcescens*, and *Enterococcus* spp. [64, 65]. Furthermore, *Serratia* spp. produces antibiotics [66], while *P. aeruginosa* produces hemagglutinins [67] and siderophores [68], and *Pseudomonas fluorescens* produces an anti-*T. cruzi* factor [69]. Finally, pigments such as prodigiosin, which is toxic to *P. falciparum* [70] and *T. cruzi* [62], are produced by gram-negative bacteria such as *Serratia* spp. and *Enterobacter* spp. [71]. In the mosquito-*Plasmodium* model, an enterobacterium, *Enterobacter* spp., was recently isolated in Zambia from a mosquito species resistant to infection by *P. falciparum*, and it was suggested that the anti-*Plasmodium* effect is due to the production of active oxygen by this bacterium [72]. Bacteria may also have indirect roles in insects. For example, Dong et al. [73] suggested that the anti-plasmodium effect mediated by bacteria may be due to the anti-bacterial immune response of the mosquito, potentially through the activation of its immune defenses. There is a great diversity of bacteria harbored by the tsetse fly gut that may produce themselves or trigger the production by their host of an equally large variety of compounds that could be harmful or beneficial to the trypanosome, to the coinhabiting gut bacteria, or even to the vector itself. While any of these compounds could be useful to combat the parasite and consequently the disease, it will be difficult to determine how to exploit them in practice.

### Could a combination of bacterial community members and their phageomes hosted in the gut of *Glossina morsitans morsitans* or *Glossina palpalis gambiense* concur preventing African Trypanosoma to initiate and complete their developmental program?

As discussed above, investigations on physiological, chemical or molecular interactions have been focused on targeted events, making it difficult to obtain an overview of the complex phenomena that occur in vivo. Indeed, the current research trend is aimed at metabolomics, proteomics and genomics approaches that are more global in nature. These approaches have been made possible thanks to technological advances in: genome sequencing and annotation; the identification of both simple and complex biological molecules; and bioinformatics and biostatistics analyses.

In this respect, sequencing and annotation (although not yet complete) of the *G. m. morsitans* genome [74] represents a major step, along with the genome sequencing of trypanosomes [75–78] and symbionts [34, 79, 80]. Importantly, the sequencing of the complete genomes of other tsetse species will allow a comparative analysis (and perhaps identification) of the genes that control the tsetse susceptibility to infection by a particular trypanosome species (among other events).

Two other global approaches have been developed in recent years. The first approach consisted in the analysis of the transcriptomes of the tsetse fly and its partners (today they only include *S. glossinidius*, *Wigglesworthia*, and *T. b. gambiense*) in order to identify the molecular dialogue and the disruptions induced by trypanosome infection. The genes associated with this event are differentially expressed following infection, with reference to the expression of the same genes in uninfected or refractory flies. Some of these genes could be involved in controlling fly susceptibility or refractoriness to infection (i.e. fly vector competence). The second approach consisted in analyzing the trypanosome secretome to identify the excreted/secreted proteins (ESPs) released by the parasite into the tsetse fly gut and that may participate in its establishment. Finally, all of these approaches aim to decipher the molecular dialog occurring in vivo between the partners, in order to identify the molecular steps that could be considered as targets to inhibit or at least reduce fly vector competence.

### Major achievements of the transcriptomics approach

#### Sodalis glossinidius

The *S. glossinidius* genome comprises 2,683 genes that are distributed across one chromosome (representing 2,523 genes) and four plasmids (accounting for 91, 31, 25, and 13 genes). Analysis of *S. glossinidius* inhabiting trypanosome-infected flies revealed 176 differentially expressed genes (DEGs), which encode a large variety of proteins including type III secretion system proteins [81, 82]. The corresponding genes are overexpressed in the symbionts of refractory tsetse flies and results in the enrichment of the KEGG pathway “bacterial secretion systems”. The effective role of the type III secretion system (injectisome) has been reported in the case of *P. aeruginosa*, which secrete toxic proteins in the cytosol of target cells [83]. Moreover, an overexpression of lysis protein transcripts was recently revealed in *Sodalis* harbored by refractory flies, with a corresponding enrichment in the “cytolysis, lysozyme activity, catabolism of peptidoglycan, bacteriolytic enzyme” functions [81, 82]. In this case, the degradation products of the bacterial cell wall peptidoglycan may be involved in activation of the immune system of tsetse flies. In fact, the lysis protein transcripts were encoded by a phage genome which indicates that the symbionts were initially lysogenic and that the prophage has been activated via an unknown mechanism allowing its genes to be expressed [72, 73]. This mechanism could indirectly impact the trypanosome ability to establish infection.

#### Wigglesworthia

The *Wigglesworthia* genome comprises 673 genes. Hamidou Soumana et al. [84] determined that more than 200 genes are differentially expressed when the symbiont is hosted in infected vs. non-infected tsetse flies. Identification of the biological functions (term Gene Ontology: GO) revealed an enrichment of the GO “metabolic and binding processes” at day 3 post fly feeding on an infected meal. At day 10 post fly infection, the enriched functions were those involved in “processes of development, morphogenesis and cellular networks processes”. At day 3 post fly feeding, *Wigglesworthia* genes encoding GroEL and GroES chaperones, non-coding RNAs, proteins involved in the transport of bacterial toxins, and proteins involved in thiamine synthesis were all under-expressed. Genes encoding GroEL and GroES chaperones were nevertheless overexpressed in the symbionts from flies at day 10 post infected meal ingestion [84]. These chaperones could function as trypanosome toxins, as shown for *Enterobacter aerogenes* (a symbiont of the antlion), which produces a toxic chaperone that paralyzes the antlion’s prey [85]. Finally, we note that from a general point of view, the expression of a given gene may vary considerably with infection time.

#### The first insights from comparative analysis of Glossina morsitans morsitans and Glossina palpalis gambiense genomes provide promising directions

In 2014, given the absence of any sequenced or annotated *G. p. gambiensis* genome, investigations have had to rely on a RNA-seq de novo assembly approach. This approach was successful in identifying DEGs in infected vs. non-infected flies on days 3 (1,373 DEGs), 10 (52 DEGs), and 20 (1,025 DEGs) post feeding [86]. DEGs were either over- or under-expressed, and they included genes encoding proteins that exhibit a large variety of activities. The most represented genes were proteases (and some protease inhibitors), as well as oxidases (i.e. laccases), lectins, hydrolases (i.e. chitinases), and anti-microbial peptides/proteins (such as Pro3 protein, transferrin, mucin, attacin, cecropin, etc.). As discussed in Hamidou Soumana et al. [86], a number of these up- or down-regulated proteins could be involved in tsetse fly susceptibility or refractoriness to trypanosome infection. Several examples of mechanisms that could mediate these processes include the triggering of fly immune defenses, the hydrolysis of fly protective structures such as the peritrophic matrix, the oxidative detoxification of toxic molecules, or the pathogen recognition process. The fact that both chronic and acute forms of sleeping sickness exist in Africa, caused respectively by *T. b. gambiense* and *T. b. rhodesiense* and transmitted respectively by *G. p. gambiensis* and *G. m. morsitans*, suggests that common molecular approaches could be developed to identify common targets to fight the disease. Indeed, one recent study was conducted to determine whether the *G. m. morsitans* genome carries any genes that are orthologous to DEGs in *G. p. gambiensis*. Specifically, the RNA-seq de novo assembled sequences from *G. p. gambiensis* were first mapped onto the *G. m. morsitans* genome in order to detect *G. m. morsitans* genes that are orthologous to *G. p. gambiensis* genes, with a special focus on the DEGs. Next, corresponding genes were annotated with respect to various databases. This approach revealed that around 50% of the *G. p. gambiensis* DEGs have orthologous genes in the *G. m. morsitans* genome [87]. Most of the *G. p. gambiensis* DEGs from this study that were considered to be of potential interest to an anti-vector strategy had a heterologous gene in *G. m. morsitans*. This list includes (but is not limited to) genes that encode proteases, chitin binding proteins, factors involved in fly immunity or antimicrobial peptide production (such as Pro3 protein), transferrin, mucin, attacin, and cecropin.

### Investigating trypanosome transcriptomics and proteomics

Recently, the transcriptomes of infected *G. p. gambiensis* flies and the *T. b. gambiense* that they harbor were collectively analyzed and the corresponding genes were annotated [86]. As observed elsewhere, the genes encoding proteases were the predominant group (including aminopeptidases, aquaporin, aspartyl-peptidases, calpain, metalloproteases, and serine proteases), along with genes encoding protease inhibitors (such as cysteine peptidase inhibitor). Interestingly, genes encoding enzymes involved in proline metabolism (such as proline oxidase and delta-1-pyrroline-5-carboxilate dehydrogenase) and the translationally controlled tumor protein (TCTP) were expressed. All of these proteins (and many others not cited here) were found to be excreted / secreted in vitro by trypanosomes when cultivated in a secretion-stimulating medium. Here, the proteases were highly predominant in the secretomes of procyclic forms of either Tbg or *T. b. brucei* [88], as well as in the bloodstream strains of *T. b. gambiense* [89].

Finally, it must be noted that TCTP was also characterized in the secretomes of diverse Tbg forms. This protein is ubiquitously distributed in eukaryotes [90] and appears to play important roles in many biological processes such as the cell cycle, apoptosis, embryo development, cell proliferation, and stress responses [91–96]. Recent study has demonstrated that TCTP also acts on bacteria isolated from the tsetse gut, and is able to modulate their growth rate in vitro [97].

### Evidence that phages and other viruses could regulate the tsetse fly microbiome composition and thus modulate fly vector competence

One recent review has provided a comprehensive of how bacteriophages could regulate bacterial communities (with consequences on human health), and how the microbial ecosystem functions [98]. This review raises the question of whether what is known about human-microbe relationships could be extended to microbe-insect relationships (and particularly the tsetse fly). The role of phages in modulating interactions between pathogens or pests and their hosts has often been reported. This occurs, for example, in aphids harboring the optional endosymbiont *Candidatus Hamiltonella defensa*, which enables the host to survive when attacked by a parasitoid wasp [99]. In fact, different strains of *H. defensa* that vary in their host protective level have been characterized, which was shown to depend on the presence of symbiont strains infected by bacteriophage, *Acyrthosiphon pisum* secondary endosymbiont (APSE) in a lysogenic phase [100]. Aphids hosting the *H. defensa* symbiont infected with APSE are significantly more resistant to parasitoid wasps than those that host the uninfected symbiont. The host protection was shown to be due to the production of a toxin encoded by the bacteriophage genome that is directed against eukaryotes which, in this particular case, is killing wasp larvae [101–103].

Three observations about tsetse flies require further discussion: the fact that *S. glossinidius* significantly favors fly infection by trypanosomes [45]; the fact that phage virions have been identified sporadically in cultures of *S. glossinidius* isolated from *G. m. morsitans* [104]; and the fact that in *S. glossinidius* from flies refractory to trypanosome infection, several genes belonging to bacteriophage genomes were highly over-expressed as compared to *S. glossinidius* from trypanosome infected (and thus susceptible) flies [81]. As discussed earlier in this review, this indicates that *Sodalis* is lysogenic and that the prophage carried by this symbiont within the refractory fly has been activated. This allows expression (using the bacterial biosynthetic machinery) of the phage genes involved in phage nucleic acid replication, capsid protein biosynthesis, and, importantly, in bacterial cell wall peptidoglycan hydrolysis, whose degradation products are effective stimulators of insect immune defenses [105]. The authors of this study concluded that the *Sodalis* phage could reduce tsetse fly infection by trypanosomes, in terms of stimulating tsetse immune defenses and by reducing the symbiont density in the tsetse gut. This latter effect is in line with previous observations that flies with a higher *Sodalis* load are more susceptible to trypanosome infection than others [105]. It should be noted that Welburn and Maudlin [106] refer to the bacterium as a “*Rickettsia*-like organism”; this “organism” was later identified as *S. glossinidius* [107]. Currently, the mechanism that induces prophage activation in vivo within the tsetse gut remains unresolved.

Besides, Salivary Gland Hypertrophy Virus (SGHVs) has been identified that infect *Glossina* sp. (GpSGHV). It is the type-species of the *Hytrosaviridae* family of insect viruses [108]. The virus replicates in the salivary glands, causes the hypertrophy of this organ and induces reproductive dysfunction [109, 110], thus impairing mass production of sterile male tsetse flies in the frame of the Sterile Insect Technique (SIT strategy) to control sleeping sickness. Peacock et al. [111] observed an association between salivary gland hypertrophy symptoms and trypanosome colonization of *G. pallidipes* salivary glands; several authors suggested viral infection to reduce the immune defenses in this organ, thus favoring its susceptibility to trypanosome infection [109, 112, 113]. In addition, Kariithi et al. [114] reported the absence of *Wolbachia*, in flies displaying salivary gland hypertrophy symptoms; this may also affect tsetse fly infection.

## Brief review of the methods /approaches for generating *Glossina morsitans morsitans* and *Glossina palpalis gambiense* populations engineered to durably replace the native tsetse flies perpetuating in distinct ecosystems

The current state of knowledge regarding fly/microbiome/trypanosome interactions suggests that tsetse flies could be made refractory to trypanosome infection. In this section we review the most promising methods to potentially accomplish this: mutagenesis, paratransgenesis, and resistance induction by intestinally residing bacteria.

### Mutagenesis: The CRISPR/Cas9 system

Recent years have seen the development of a novel molecular tool for genome editing known as Clustered Regularly Interspaced Short Palindromic Repeat (CRISPR). Regarding insect research, CRISPR has been especially applied to *Drosophila melanogaster*, *Bombyx mori*, and *Aedes aegypti*. CRISPR generates RNAs from bacterial genomes that, when associated to nucleases, will guide these enzymes to complementary sequences of foreign DNA (such as DNA from invading pathogens); when cleaved, the foreign DNA will induce deletions or insertions in the corresponding genome (for a comprehensive recent review, refer to [115]). The CRISPR approach is expected to become a powerful tool for directed mutagenesis, with useful applications in functional analysis and introducing genes of interest (such as toxins targeted at invading pathogens) into the genome of a given organism. Thus, besides its use as a tool for genetic engineering, CRISPR could be used to fight pests and pathogens, including vector-borne diseases. There are however some restrictions. For a practical application against pathogens in the field, the host that has been genetically transformed by CRISPR must be able to transmit the beneficial character to the next generation, in order to progressively replace the susceptible population by a resistant one. Thus, for sexually reproducing organisms, the mutation must target the germ cells. However, the fact that tsetse flies are viviparous (i.e. the development of the embryos take place in utero) indicates that manipulation by CRISPR is currently not feasible (at least very difficult to perform as it would need germ-line transformation in utero).

### Paratransgenesis

As opposed to genetic transformation of the tsetse fly target organism, paratransgenesis makes use of a compound of interest (such as toxins, immune response-stimulating molecules, gene repressors/activators, or enzyme activity inhibitors/stimulators) that is introduced in vivo into the fly by a microorganism which after being suitably selected or engineered, will be injected into the tsetse gut. This approach was suggested by Rio et al. [116], who considered the secondary symbiont *S. glossinidius* to be well-adapted for such a “paratransgenesis” strategy. This choice is supported by five lines of evidence: the symbiont grows in the fly gut and hemolymph as do trypanosomes [42]; it can be isolated, cultivated in vitro, and genetically transformed [42, 107, 117, 118] as it was shown by De Vooght et al. [118], when introducing a functional anti-trypanosome nanobody; it can be reintroduced into the fly [119]; it is most frequently transmitted maternally to the offspring [33, 120], despite the recent demonstration of paternal transmission during mating [121]; and finally, due to the large-scale erosion of its genome [122, 123], *Sodalis* is metabolically dependent on its tsetse fly host, suggesting that no gene flow towards any other organisms will occur once the tsetse fly harboring the modified symbiont is distributed in HAT foci.

### Tsetse fly refractoriness induced by intestinally residing bacteria

In insects, gut bacteria can affect interactions with a parasite. For example, the level of susceptibility in bumble bees to the *Crithidia bombi* parasite is determined by the specific composition of the host intestinal microflora [124]. The tsetse fly microbiome is composed of diverse bacteria that can vary greatly according to the geographic/ecological situation of the investigated HAT foci. However, no statistically significant association between microbiome composition and the environment has been demonstrated yet, possibly due to overly restrictive sampling (i.e. the number of flies and foci, a lack of environmental contrasts, etc.). Thus, a broad sampling campaign is currently being performed on the vectors of malaria (*anopheles*) and sleeping sickness (tsetse flies), which associates networks of scientific partners from countries endemic or not for both diseases. The aims include: identifying all bacteria hosted by either the mosquitoes or the flies; determining whether there is an association between microbiome composition, vector infection status, and the environment; and determining if there is an association with a specific bacterium, or a restricted number of bacterial species. Once any such bacteria are identified, their physiological characteristics will need to be determined, especially regarding their potential involvement in the fly infection process (and thus vector competence) and their transmission from one fly generation to the next. Thus, the use of such natural intestinal “anti-parasitic” bacteria could be a suitable alternative to paratransgenesis.

### Methods to disseminate refractory tsetse flies in the field

Once there are available tsetse flies that harbor transformed symbionts or anti-parasitic bacteria, it will be necessary to determine how to effectively and efficiently disseminate these flies in HAT foci of interest. One classical approach is that used in the so-called “sterile insect technique (SIT) strategy” relying on the release in a very large number of such engineered sterile males in the trypanosome infected foci. However, this would require the massive and periodic production and release of flies in order to overcome the natural female fly population of the target focus (and perhaps females coming from neighboring foci as well).

One promising alternative approach suggested by Alam et al. [56] takes advantage of the induction produced by *Wolbachia* under specific conditions, resulting in a strong cytoplasmic incompatibility in the host. Briefly, this incompatibility occurs when a *Wolbachia*-negative female tsetse fly (W-) mates with a *Wolbachia*-positive male tsetse fly (W+), resulting in embryonic development failure and the absence of any progeny. In contrast, crossing a (W+) female tsetse to a (W+) or (W-) male results in viable, fertile progeny that are more numerous than if a (W-) female is crossed with a (W-) male. Thus, in accordance with their reproductive advantage, the dissemination in a focus of (W+) females that host the modified *Sodalis* symbiont will result in the progressive replacement of the natural population by a population of modified tsetse flies refractory to trypanosome infection. According to Alam et al. [56], a dissemination corresponding to 10% of the natural population should lead to the replacement of 90% of this population within only 2 years.

### Before concluding, some data highlighting the presence, in the dermis of healthy individuals, of *Trypanosoma brucei gambiense* transmissible to the tsetse flies

Biologically relevant experimental studies conducted in laboratory mice to which were delivered *Trypanosoma brucei brucei* recently allowed documenting the prolonged presence in the mouse dermis of both slender and stumpy developmental stages. Then, they conducted an extensive histological analysis of archived skin biopsies collected from human individuals without a history of HAT enrolled in the in the Democratic Republic of the Congo as part of a screening programme for river blindness. Out of several thousands skin biopsies 0.5% trypanosomes were detected in the extracellular matrix of the vascularized dermis, namely the compartment where the pool blood feeding tsetse flies are known to sample their blood meal from the blood pool they generate: the motile stumpy developmental stage population is expected to be sampled from this blood pool [125, 126].

The occurrence of asymptomatic infections is actively monitored and documented [127]; their impact should not be underestimated. However, continued efforts are needed to refine methods to detect these infections and to evaluate the rate of transmission, to pre-adapted flies, of African Trypanosomes from the dermis of mammals from which these flies sample their blood meals.

## Conclusions

Despite a decrease in the number of diagnosed cases, sleeping sickness continues to inflict a heavy burden on the people of Africa living in or near trypanosome-infested areas. For this reason, new research projects are continually and actively developed to control and possibly eradicate the disease, with financial support from a variety of agencies (including the WHO, PATTEC, IAEA, various African, European and American Research Institutes, the Departments of Health in afflicted African countries, and the Bill and Melinda Gates Foundation).

This review has focused on diverse approaches useful for identifying molecular or bacterial targets that may make it possible to render the tsetse fly refractory to trypanosome infection, in order to block parasite transmission to humans and animals. Transcriptomic analyses of the tsetse fly and its *Sodalis glossinidius* and *Wigglesworthia* partners have provided a wealth of information on the genes that are associated with tsetse susceptibility or refractoriness to trypanosome infection. However, this only represents part of the final goal. Indeed, it is still necessary to verify the results recorded under artificial infection conditions with flies sampled in the field. Most importantly, targets must be selected and tested for their relevance and effectiveness through functional analyses. Similarly, after identifying the bacteria that compose the fly microbiome, suitable candidates must be selected and tested for their possible effectiveness on fly vector competence. Finally, extensive continuing research is necessary in order to obtain tsetse flies that are refractory to infection, so that their distribution in the field can render the overall tsetse population refractory. In addition, this method will present the advantage to preserve the pre-existent environmental biodiversity as the objective will consist in the replacement of the susceptible (or possibly susceptible) individuals by their refractory counterparts, not in the elimination of the fly populations. The control (and possible eradication) of HAT is the final goal, in order to protect people from the devastation of sleeping sickness. However, achieving this objective will require the continuing deployment of a multitude of approaches. In this spirit, it should also be kept in mind that the results obtained from sleeping sickness investigations could be used to help decipher the mechanisms of other vector-borne diseases.

## Abbreviations

AAT: animal African trypanosomiasis
APSE: *Acyrthosiphon pisum* secondary endosymbiont
CRISPR: Clustered Regularly Interspaced Short Palindromic Repeat
DEGs: differentially expressed genes
EP protein: Glutamic Acid – Proline protein
ESPs: excreted/secreted proteins
GO: Gene Ontology
GpSGHV: *Glossina* sp. Salivary Gland Hypertrophy Virus
HAT: human African trypanosomiasis
Imd: Immune Deficiency
PATTEC: Pan-African Tsetse and Trypanosomiasis Eradication Campaign
PGRP-LB: peptidoglycan recognition protein LB
SGHVs: Salivary Gland Hypertrophy Virus
SIT: Sterile Insect Technique
TCTP: translationally controlled tumor protein
